# Single-molecule quantification of 5-hydroxymethylcytosine for diagnosis of blood and colon cancers

**DOI:** 10.1186/s13148-017-0368-9

**Published:** 2017-07-14

**Authors:** Noa Gilat, Tzlil Tabachnik, Amit Shwartz, Tamar Shahal, Dmitry Torchinsky, Yael Michaeli, Gil Nifker, Shahar Zirkin, Yuval Ebenstein

**Affiliations:** School of Chemistry, Center for Nanoscience and Nanotechnology, Center for Light-Matter Interaction, Raymond and Beverly Sackler Faculty of Exact Sciences, Tel Aviv University, Tel Aviv, Israel

**Keywords:** CRC, Colon cancer, Blood cancer, Chronic lymphocytic leukemia, Multiple myeloma, Acute lymphoblastic leukemia, Single-molecule, 5-hydroxymethylcytosine, 5hmC

## Abstract

**Background:**

The DNA modification 5-hydroxymethylcytosine (5hmC) is now referred to as the sixth base of DNA with evidence of tissue-specific patterns and correlation with gene regulation and expression. This epigenetic mark was recently reported as a potential biomarker for multiple types of cancer, but its application in the clinic is limited by the utility of recent 5hmC quantification assays. We use a recently developed, ultra-sensitive, fluorescence-based single-molecule method for global quantification of 5hmC in genomic DNA. The high sensitivity of the method gives access to precise quantification of extremely low 5hmC levels common in many cancers.

**Methods:**

We assessed 5hmC levels in DNA extracted from a set of colon and blood cancer samples and compared 5hmC levels with healthy controls, in a single-molecule approach.

**Results:**

Using our method, we observed a significantly reduced level of 5hmC in blood and colon cancers and could distinguish between colon tumor and colon tissue adjacent to the tumor based on the global levels of this molecular biomarker.

**Conclusions:**

Single-molecule detection of 5hmC allows distinguishing between malignant and healthy tissue in clinically relevant and accessible tissue such as blood and colon. The presented method outperforms current commercially available quantification kits and may potentially be developed into a widely used, 5hmC quantification assay for research and clinical diagnostics. Furthermore, using this method, we confirm that 5hmC is a good molecular biomarker for diagnosing colon and various types of blood cancer.

**Electronic supplementary material:**

The online version of this article (doi:10.1186/s13148-017-0368-9) contains supplementary material, which is available to authorized users.

## Background

5-Hydroxymethylcytosine (5hmC) is a modified form of the DNA base cytosine. 5hmC is produced through oxidation of 5-Methylcytosine (5mC) by the oxygenase ten-eleven translocation (TET) enzyme family [1–5]. In addition to serving as an intermediate of the demethylation process, 5hmC is presumed to have an important role in gene expression and gene regulation [5–9]. In respect to cancer, loss of 5hmC is observed across a wide spectrum of malignant tumors, including colorectal, breast, and prostate cancers, which display drastic reduction of the 5hmC level compared with benign and pre-malignant tumors [10–13]. As opposed to transformations in DNA methylation, such as hypermethylation of the colorectal cancer (CRC)-associated miR-137 locus [14], 5hmC does not have to be mapped to specific genomic regions. Due to its relatively low level in genomic DNA, even local changes in 5hmC content result in significant global modulations in 5hmC content. From a diagnostic perspective, this is reminiscent of protein and RNA biomarkers, potentially simplifying its use in cost-effective diagnostic applications.

Several methods currently exist for the detection and quantification of 5hmC, yet none of these methods fully meets the requirements of advanced epigenetic-based diagnostics. HPLC coupled to mass spectrometry (HPLC-MS) was the first method used to globally assess cytosine, 5mC, and 5hmC in Purkinje and granule cells [2]. This “gold standard” method is highly quantitative but requires specific expertise and expensive equipment. In addition, large amounts of starting material are needed when analyzing tissue with low 5hmC levels such as blood. Immuno-based assays are the most commonly used, including dot blot [12], immunohistochemical assays [11, 15], and enzyme-linked immunosorbent assays (ELISA) [16, 17], utilizing commercially available antibodies specific to 5hmC. The detection limit of these assays is usually higher than ~0.03% 5hmC/dNTPs, a fact that limits their utility in analysis of many human cancer tissues. Additionally, these assays only deliver relative 5hmC levels, while for absolute measurements, a calibration curve must be established. Another method for detecting 5hmC utilizes the T4 phage enzyme β-glucosyltransferase (β-GT) [18, 19]. β-GT catalyzes the attachment of β-d-glucosyl residues from uridine diphosphoglucose (UDP-Glu) to the hydroxyl group of 5hmC. This conversion allows addressing 5hmC via the sugar attached to it. In previous work, we introduced a two-step labelling reaction based on the β-GT enzyme [20–22]. By replacing its original cofactor with uridine diphosphate-6-azide-glucose (UDP-6-N_3_-Glu), 5hmC is labelled with a reactive azide group which can then be further reacted via a copper-free Huisgen cycloaddition (click reaction) [23], where the azide group is linked to a fluorescently labelled strained alkyne, dibenzocyclooctyne-Cy5 (DBCO-Cy5). The reaction results in 5hmC residues tagged with fluorescent reporter molecules. While this method provided accurate and high-throughput quantification of 5hmC in multi-well plates [22], large amounts of DNA starting material were required (~6 μg) and the detection limit was insufficient for the quantification of 5hmC in tissues with extremely low 5hmC level, such as blood and cancer tissues. In order to enhance the detection sensitivity and to reduce the amount of starting material, we utilize an ultra-sensitive single-molecule approach by which fluorescent 5hmC marks are directly visualized and counted on individual chromosomal fragments. Here, we demonstrate the applicability of this approach for the detection of colon cancer and several blood cancer types and emphasize the potential of 5hmC as a biomarker for CRC, providing significantly larger sensitivity and dynamic range relative to recently reported studies [10, 11, 24–26].

## Methods

### Patients and study design

#### Colorectal cancer

Commercially available DNA extracted from polyps and adjacent tissue was purchased for analysis (BioServe). We tested DNA samples from seven healthy individuals and seven CRC patients. All tissue samples were taken from men and women 30–65 years old.

#### Blood cancer

Commercially available DNA extracted from human peripheral blood cells and frozen peripheral blood cells were purchased for analysis (BioServe, HemaCare). We tested DNA samples from eleven healthy individuals, eight chronic lymphocytic leukemia (CLL) patients, four multiple myeloma (MM) patient, and three acute lymphocytic leukemia (ALL) patients, taken from men and women 60–85 years old.

DNA was extracted from peripheral blood cells using the GenElute™ Mammalian Genomic DNA Miniprep Kit (Sigma).

For the purposes of the single-molecule assay, both colon and blood DNA were handled with care throughout the entire procedure in order to minimize fragmentation of DNA molecules.

### 5hmC labelling procedure

5hmC was fluorescently labelled in a two-step chemical labelling procedure [22]. Three hundred nanograms of genomic DNA was mixed with 3 μl of buffer 4 (NEB), 0.5 μl of UDP-6-N_3_-Glu (0.3 mM, [20]), 2 μl of T4 bacteriophage β-glucosyltransferase (T4-BGT, NEB) and ultrapure water to a final volume of 30 μl. The reaction was mixed and incubated overnight at 37 °C. On the following day, 0.15 μl of DBCO-Sulfo-Cy5 (10 mM, Jena Biosciences) was added to the mixture, followed by a second overnight incubation at 37 °C (click reaction). Upon completion of the labelling procedure, residual fluorophore was removed by drop dialysis (0.1 μm, Millipore) against 300 ml of TE buffer for 2–3 h. Alternatively, samples were washed by ethanol precipitation or by magnetic beads (MagVigen DNA Select Kit, NVIGEN). Samples were stored at 4 °C until analyzed.

### Preparation of activated glasses for stretching DNA

Glass surfaces for DNA extension were prepared according to [21] with minor modifications. In short, standard glass cover slides were first cleaned by immersing them overnight in 2:1 (*v*/*v*) mixture of nitric acid (70%) and hydrochloric acid (37%), respectively. On the following day, the slides were washed extensively by using ultrapure water and ethanol and dried under nitrogen stream. Dried slides were inserted into a 300 ml aqueous solution containing 1200 μl *N*-trimethoxysilylpropyl-*N*,*N*,*N*-trimethylammonium chloride (Gelest) and 180 μl vinyltrimethoxysilane (Gelest), then incubated for 17.5 h at 65 °C with mild shaking. Slides were then washed extensively by using ultrapure water and ethanol and stored in ethanol at 4 °C until use.

### Single-molecule imaging

Labelled samples were diluted 1:100–1:300 in HEPES-DTT buffer (100 mM HEPES, 200 mM DTT, Sigma) and were stained with 130 nM YOYO-1 (Invitrogen) DNA intercalating dye. Eight microliters of each sample were extended on a glass slide by placing the solution at the interface of an activated coverslip and a clean microscope slide. The extended DNA molecules were imaged with a fluorescence microscope (TILL photonics GmbH) using an Olympus UPlanApo 100X 1.3 NA oil immersion objective. Each image was composed of two colors, the YOYO-1 and the Cy5 fluorophores, and was therefore imaged with the appropriate filters (485/20 and 650/13 bandpass excitation filters, 525/30 and 684/24 bandpass emission filters, for YOYO-1 and Cy5, respectively). Images were acquired by a DU888 EMCCD (Andor technologies) with an EM gain setting of 300 and exposure times of 200 and 3000 ms for YOYO-1 and Cy5, respectively.

### Data analysis

Images were analyzed using in-house image processing software. The total DNA length of each molecule was calculated, and the number of collocalized 5hmC labels on each molecule was counted. By dividing the total amount of 5hmC fluorescent spots that were found in an image set by the total DNA length, a measurement of the 5hmC level was accurately calculated for each sample.

Statistical analysis of the data was performed automatically using custom software that continuously plots the 5hmC level as a function of the total amount of DNA acquired (calculated as number of dots/total DNA length in pixels). The graph helps assessing the required amount of data needed for accurate 5hmC measurement; upon sampling more data, the 5hmC level readout stabilizes, finally reaching a plateau, indicating sufficient data was sampled. Additionally, the software randomly divides the total data of a specific sample into two groups containing similar amount of DNA and measures the %5hmC in each group. The standard deviation between 5hmC levels in the different groups was calculated and was verified to be less than 10%.

In order to account for variations between experiments, a well-characterized calibration sample was used side by side with the analyzed samples for normalization purposes. Finally, measured 5hmC levels were calibrated to report on the percentage of 5hmC bases out of the total dNTPS. This was done by applying a conversion factor accounting for experimental parameters such as labelling efficiency and the DNA stretching factor over activated glass slides. The level of 5hmC was validated using HPLC-MS (see Additional file 1: Figure S2).

## Results

**Fig. 1:**
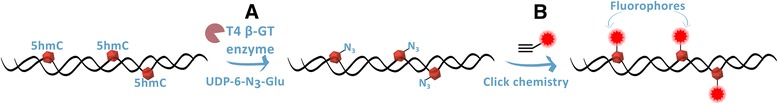
Schematic representation of the two-step 5hmC labelling reaction. **a** First, T4 β-GT enzymatic glucosylation of 5hmC with UDP-6-N_3_-Glu is performed. **b** Next, click reaction between the N_3_ group and the fluorescently labelled alkyne DBCO-Cy5 is performed. This two-step reaction results in fluorescently labelled 5hmC

**Fig. 2:**
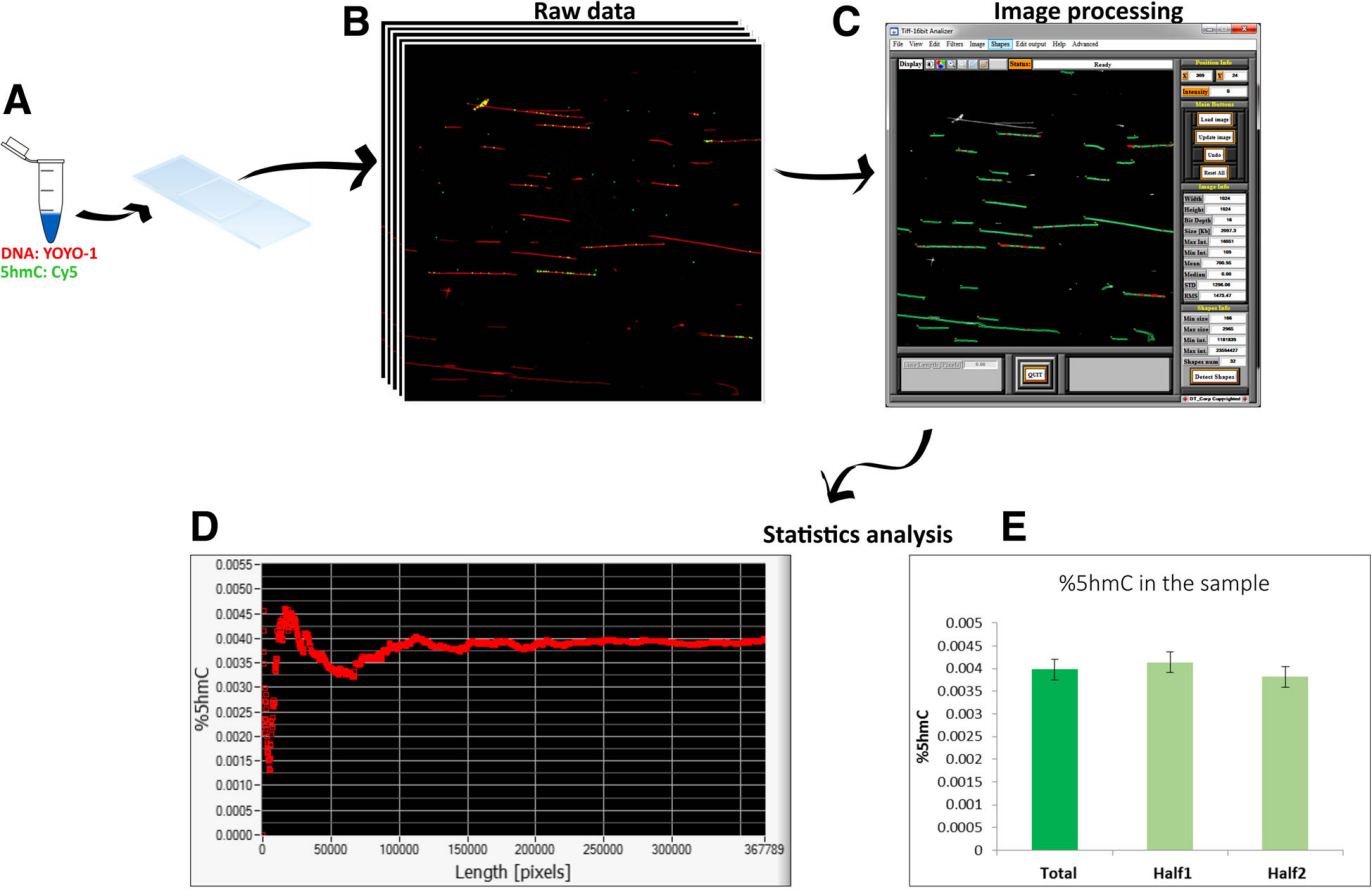
Illustration of sample imaging and analysis process. **a** Two-colored DNA molecules are stretched on glass slides. **b** Multiple fluorescence microscope images are obtained per sample, showing both YOYO-1 labelling of the entire molecule (*red*) and the Cy5 labelling of 5hmC-modified nucleobases (*green*/*yellow dots*). **c** User interface of the image processing software, where the length of the DNA molecules is measured (*green*) and collocalized 5hmC (*red dots*) is detected and subsequently quantified relative to DNA length. **d** Output of the statistics tool, showing the total length of DNA (in pixels, *X* axis) and the percentage of 5hmC relative to DNA (%5hmC, *Y* axis). **e** Comparison of %5hmC of the total sample data with two randomly divided subsets of this data which contain similar amount of DNA

5hmC residues in DNA samples extracted from colon tissue and peripheral blood cells were fluorescently labelled by the two-step labelling reaction (Fig. 1). Labelled samples were then stretched on glass slides and imaged using a fluorescence microscope. DNA molecules appear as extended red lines dotted with yellow/green spots indicating 5hmC sites (Fig. 2a, b). Finally, 5hmC level in the samples was determined using image analysis (Fig. 2c), and the statistical validity of the data was assessed (Fig. 2d, e).

The main advantage of the above described assay is its extreme sensitivity, limited only by the diffraction limit of the optical imaging system which is being used. A standard fluorescence microscope equipped with an oil immersion objective will typically allow distinguishing between two adjacent fluorophores separated by 200–300 nm (300–1000 bp apart). Since close by 5hmC residues may appear as a single fluorescent spot, we quantified the statistical distribution of clustered 5hmC sites by measuring photobleaching steps (see Additional file 1: Figure S1). The majority of the analyzed 5hmC sites contained a single fluorophore, implying that isolated observable marks are comprised of mostly single 5hmC residues. Using the full photobleaching step distribution enabled us to correct for 5hmC clustering in the calibration process. The reproducibility of this assay was verified by repeating the labelling and imaging process two to four times for each of the analyzed samples, with an average standard deviation of 5% per sample (see Additional file 1: Tables S1, S2).

### Colorectal cancer

**Fig. 3:**
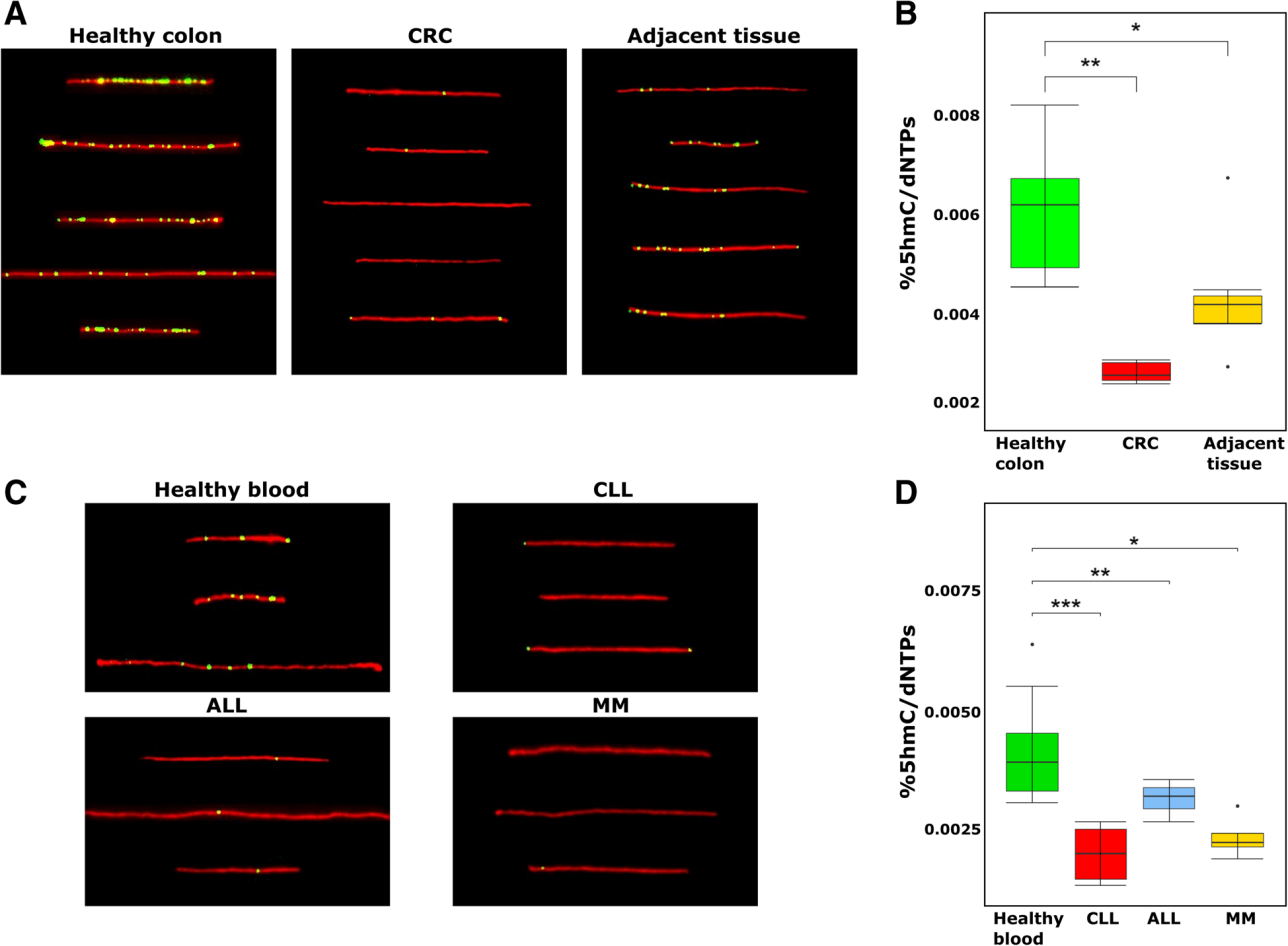
**a** Fluorescence microscopy images of representative DNA molecules (*red*) with 5hmC labelling (*green*/*yellow*) extracted from healthy colon tissue, CRC, and adjacent colon tissue taken from the same patient. **b** Box plot comparison of 5hmC level as calculated from imaged molecules of healthy colon (*n =* 7), CRC (*n =* 7), and adjacent colon tissue (*n =* 7). **c** Fluorescence microscopy images of representative DNA molecules (*red*) with 5hmC labelling (*green*/*yellow*) extracted from healthy, CLL, ALL, and MM peripheral blood cells respectively. **d** Box plot comparison of 5hmC level as calculated from imaged molecules of healthy (*n =* 11), CLL (*n =* 8), ALL (*n =* 3), and MM (*n =* 4) peripheral blood cells

Colon biopsies are commonly obtained during regular screening colonoscopy procedures. The small amount of sample needed to perform this assay allowed us to assess 5hmC levels using down to 50 ng of DNA. Significant decrease in 5hmC level is observed in CRC tissue (0.0028% 5hmC/dNTPs, *n* = 7) compared with healthy colon tissue (0.0059% 5hmC/dNTPs, *P* < 0.0005, *n* = 7). In addition, the 5hmC level in adjacent colon tissue taken from the same CRC patients (0.0043% 5hmC/dNTPs, *n* = 7) is higher than that found in tumor tissue, yet it is significantly lower than the level of colon tissue from a healthy individual (*P* < 0.05) indicating that staging of disease may be possible (Fig. 3a, b).

### Blood cancer

Blood is the most accessible tissue for diagnostic applications. However, blood exhibits extremely low levels of 5hmC, a fact that has made it a challenging tissue for 5hmC-based studies. The sensitivity of our method allowed us to clearly differentiate between healthy and malignant blood samples. Significant decrease in 5hmC level is observed in samples of all three types of blood cancer tested, namely CLL (0.0019% 5hmC/dNTPs, *P* < 0.0001, *n* = 8), ALL (0.0031% 5hmC/dNTPs, *P* < 0.05, *n* = 3), and MM (0.0023% 5hmC/dNTPs, *P* < 0.005, *n* = 4), as compared with healthy blood samples (0.0042% 5hmC/dNTPs, *n* = 11, Fig. 3c, d). We note that peripheral blood samples from lung cancer and CRC patients were also analyzed. No correlation was found between 5hmC levels in these blood samples and the state of disease, indicating that, as expected, the use of 5hmC as a biomarker for cancer is tissue-specific.

### Assessment of commercial kits

In order to compare our method with existing techniques, we have tested two commercial ELISA kits, which are of the most cited immuno-based methods for 5hmC quantification (Quest 5-hmC™ DNA ELISA Kit (ZYMO research) and MethylFlash™ Hydroxymethylated DNA Quantification Kit (EPIGENTEK)).

**Fig. 4:**
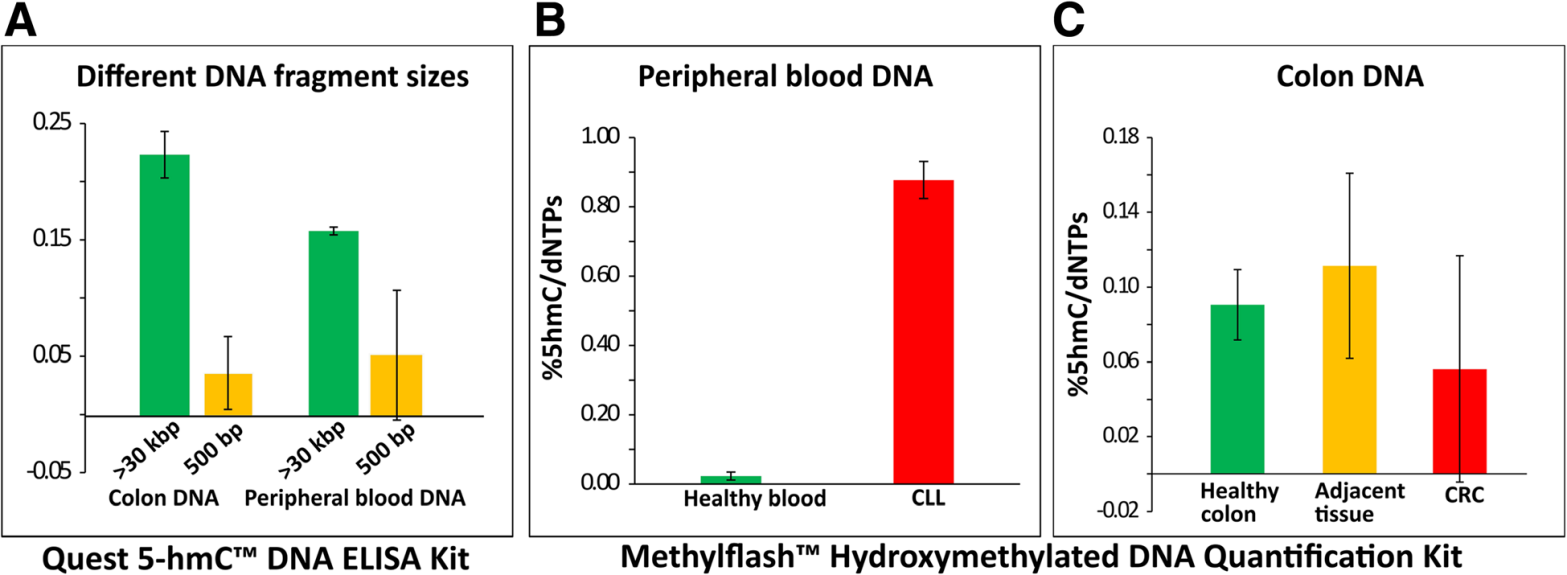
Measurement of 5hmC using commercial ELISA kits. **a** Quest 5-hmC™ DNA ELISA Kit. Bar graphs representing 5hmC levels in colon and peripheral blood DNA samples measured at two average fragment lengths. DNA fragments >30 kbp (*green bars*) and DNA fragments 500 bp in length (*yellow bars*). Different fragment lengths for the same DNA sample produced significantly different results. **b**, **c** MethylFlash™ Hydroxymethylated DNA Quantification Kit. **b** Bar graph comparison of 5hmC level as calculated by the kit for healthy blood (*n =* 1) and CLL (*n =* 1). **c** Bar graph comparison of 5hmC level as calculated by the kit for healthy colon (*n =* 2), adjacent tissue (*n =* 2), and CRC (*n =* 2). Both absolute and relative 5hmC levels are inconsistent with published data and the measurements presented in this study

We found that the 5hmC levels measured by the Quest 5-hmC™ DNA ELISA Kit are extremely sensitive to the DNA fragment size in the analyzed sample. This is likely due to the use of anti-DNA antibody as a reporter (hence, longer DNA fragments result in stronger signal regardless of the 5hmC content). For comparison, we have measured two of the samples used in the single-molecule experiment (DNA extracted from adjacent colon tissue and healthy peripheral blood). The samples were analyzed as received (30 kbp average fragment length) and after fragmentation on a Covaris S220 focused-ultrasonicator instrument (500 bp average fragment length). As evident from the results presented in Fig. 4a, the 5hmC level for the same sample is reduced three to sixfold after fragmentation. These results imply that while different samples with similar fragment sizes may be qualitatively compared for their relative 5hmC content, an unbiased, quantitative measurement using this kit is limited.

The MethylFlash™ Hydroxymethylated DNA Quantification Kit has shown less sensitivity to DNA fragment length; however, the 5hmC levels obtained with this kit were inconsistent with previously published results as well as the results presented in our study, which were validated with HPLC-MS results (see Additional file 1: Figure S2). Specifically, we measured the 5hmC level of colon DNA and peripheral blood DNA samples (healthy peripheral blood (*n =* 1), CLL (*n =* 1), healthy colon (*n =* 2), adjacent tissue (*n =* 2), and CRC (*n =* 2)). The 5hmC level of the CLL sample measured with the MethylFlash kit was 20-fold higher than the 5hmC level of the healthy blood sample (Fig. 4b), most likely due to limit of detection issues. These results are inconsistent with our findings, nor are they in line with known 5hmC levels of peripheral blood tissue [27, 28]. Furthermore, the 5hmC level of colon adjacent tissue appeared higher than the 5hmC level of healthy colon tissue (Fig. 4c), in contrast to the results obtained by our assay and in disagreement with other studies [6, 10, 24].

Altogether, our findings suggest that the tested ELISA kits for 5hmC quantification have several limitations, including DNA-length dependency, low sensitivity, and poor reproducibility.

## Discussion

The loss of 5hmC is observed across a wide range of malignant tumors and can therefore be used as an informative biomarker for cancer. Here, combining 5hmC labelling and single-molecule imaging, we present a new approach for ultra-sensitive detection and quantification of 5hmC in cancer. This methodology is clearly suitable for the detection of colorectal and blood cancers. The observed differences in 5hmC level between healthy and cancerous tissues in both colorectal and blood cancers are reproducible and significant, enabling a facile and reliable distinction between the two states. These results are in line with recent studies reporting a decrease in 5hmC level in various cancer types [10, 11, 25]. Attempts to detect lung cancer and CRC using peripheral blood tissue were unsuccessful, reaffirming the fact that 5hmC is a tissue-specific biomarker. The key advantages of the reported method are its ultra-sensitive detection capability, well below the 5hmC detection limit of most reported assays [16, 17, 22, 29], as well as the fact that it provides absolute quantitative results. In addition, we were able to use starting material amounts down to 50 ng DNA per sample. In order to image a single sample, only ~8 ng of DNA per glass slide is required, and merely a few hundred femtograms of DNA were sampled in order to achieve reliable results. These results imply that input material amounts could potentially be further decreased by optimizing the labelling protocol, potentially allowing future analysis of liquid biopsies and tissue sections. In comparison, HPLC-MS analysis, which is also quantitative with similar sensitivity to the presented assay, typically requires about 0.5–2 μg of DNA as a starting material [24, 28, 30] and requires expertise and expensive equipment. The commonly used ELISA and dot blot assays require 300 ng–1 μg yet provide lower detection and quantification capabilities, which cannot adequately address the 5hmC levels in cancer and blood tissues.

Colorectal cancer is the fourth most common cancer-related cause of death, with over half a million deaths per year worldwide [31]. A major bottleneck in the process of colorectal sample analysis is the pathological examination, which is carried out manually. Our method may assist pathologists in cases where traditional examination is inconclusive (such as premalignant or adjacent tissue) by adding another layer of quantitative molecular information. The relatively high dynamic range of the CRC results and the fact that an absolute measure of 5hmC content is provided may allow staging of the disease and may even be indicative as a measure for response to therapy.

The most accessible tissue for medical analysis is peripheral blood; however, the level of 5hmC in blood derived DNA is extremely low (0.004%), a fact that has limited its utility as a practical biomarker for clinical diagnostics. Due to its high sensitivity, the reported method enables the detection of minute amounts of 5hmC in blood samples and can accurately distinguish between healthy individuals and individuals suffering blood malignancies. A key factor for successful cancer prognosis is early diagnosis. By detecting the reduction of 5hmC level in peripheral blood cells, our method will potentially enable early detection of blood cancer by a simple blood test. While existing techniques are able to detect the 5hmC level found in CRC tissues [11, 25], currently no available method enables facile, accurate, and non-expensive detection of 5hmC levels as low as found in blood samples and specifically in blood cancer samples. Furthermore, our method requires only very low amounts of DNA for the determination of the 5hmC level in a sample.

## Conclusions

Here, we present a method for the detection and quantification of 5hmC in genomic DNA and demonstrate its applicability for diagnosis of colorectal cancer and several blood cancer types. This assay could potentially enable earlier diagnosis of cancer and tailored therapy.

## Additional file

Additional file 1: Additional details on analysis. (DOCX 378 kb)

## Abbreviations

5hmC: 5-Hydroxymethylcytosine
5mC: 5-Methylcytosine
ALL: Acute lymphocytic leukemia
CLL: Chronic lymphocytic leukemia
CRC: Colorectal cancer
DBCO-Cy5: Dibenzocyclooctyne-Cy5
MM: Multiple myeloma
TET enzymes: Ten-eleven translocation enzymes
UDP-6-N3-Glu: Uridine diphosphate-6-azide-glucose
