# Supplementary material for: Single-molecule quantification of 5-hydroxymethylcytosine for diagnosis of blood and colon cancers
Source: Clin Epigenetics. 2017 Jul 14;9:70. doi: 10.1186/s13148-017-0368-9 (PMC5512773; doi:10.1186/s13148-017-0368-9)
Supplement: Additional file 1: — Additional details on analysis. (DOCX 378 kb) [file 13148_2017_368_MOESM1_ESM.docx]

**Additional file 1: Additional details on analysis**

**Colon samples analysis**

A total of 21 samples of human colon DNA (seven healthy colon, seven CRC and seven adjacent tissue samples) were labelled and analyzed. Data acquired included five independent experiments. In each experiment, one sample served as a calibration sample, intended to allow the normalization of the experiments to one another. The standard deviation of %5hmC/dNTPS for a specific sample was calculated as described in the main text.

Table 1S contains the list of colon samples analyzed in the five experiments, including the total amount of DNA sampled (in base pairs) and the 5hmC level of the sample.

| **Type** | **Sample ID** | **Total length sampled [bp]** | **%5hmC/dNTPs** | **Standard deviation** |
| --- | --- | --- | --- | --- |
| Healthy colon | 301001 | 3.72x10^7^ | 0.0079 | 9.9X10^-5^ |
| Healthy colon | 301002 | 1.53X10^8^ | 0.0043 | 6.4X10^-5^ |
| Healthy colon | 301003 | 1.20X10^8^ | 0.0047 | 2.0X10^-4^ |
| Healthy colon | 301013 | 3.44X10^7^ | 0.0061 | 4.5X10^-5^ |
| Healthy colon | 102035 | 2.15X10^8^ | 0.0050 | 1.9X10^-5^ |
| Healthy colon | 101436 | 2.00X10^7^ | 0.0068 | 4.8X10^-4^ |
| Healthy colon | 102094 | 2.02X10^8^ | 0.0065 | 1.1X10^-4^ |
| CRC | VZAPCD05 | 1.86X10^8^ | 0.0025 | 4.5X10^-4^ |
| CRC | QFHRSD01 | 1.24X10^8^ | 0.0029 | 1.8X10^-4^ |
| CRC | TZXQUD05 | 3.40X10^8^ | 0.0025 | 1.0X10^-5^ |
| CRC | EY18LD01 | 2.63X10^8^ | 0.0032 | 2.0X10^-5^ |
| CRC | KKFKYD05 | 2.62X10^8^ | 0.0029 | 1.7X10^-4^ |
| CRC | 6XCIID01 | 9.55X10^7^ | 0.0029 | 3.9X10^-6^ |
| CRC | N9B26D01 | 9.55X10^7^ | 0.0029 | 2.8X10^-5^ |
| Adjacent tissue | VZAPCD01 | 2.78X10^8^ | 0.0029 | 1.2X10^-6^ |
| Adjacent tissue | QFHRSD05 | 3.97X10^8^ | 0.0043 | 1.0X10^-5^ |
| Adjacent tissue | TZXQUD01 | 1.68X10^8^ | 0.0036 | 4.5X10^-5^ |
| Adjacent tissue | EY18LD05 | 1.67X10^8^ | 0.0043 | 1.9X10^-4^ |
| Adjacent tissue | KKFKYD01 | 1.03X10^8^ | 0.0043 | 1.4X10^-4^ |
| Adjacent tissue | 6XCIID03 | 6.77X10^7^ | 0.0040 | 2.9X10^-5^ |
| Adjacent tissue | N9B26D03 | 2.14X10^8^ | 0.0065 | 3.3X10^-5^ |

**Table 1S**

**Blood samples analysis**

A total of 26 samples of human blood cells DNA (eleven healthy blood, eight CLL, four MM and three ALL samples) were labelled and analyzed. Data acquired included 15 independent experiments. The normalization procedure for these experiments was similar to the one described above for the colon samples analysis.

Table 2S contains the list of blood samples analyzed in the 15 experiments, including the total amount of DNA sampled (in base pairs) and the 5hmC level of the sample.

| **Type** | **Sample ID** | **Total length sampled [bp]** | **%5hmC/dNTPs** | **Standard deviation** |
| --- | --- | --- | --- | --- |
| Healthy blood | EEEGF | 9.27X10^7^ | 0.0035 | 1.4X10^-4^ |
| Healthy blood | 5FAHU | 4.55X10^8^ | 0.0037 | 4.1X10^-5^ |
| Healthy blood | 7MKZ2 | 5.41X10^7^ | 0.0040 | 3.9X10^-5^ |
| Healthy blood | EVZBM | 3.56X10^8^ | 0.0040 | 8.1X10^-5^ |
| Healthy blood | DN59B | 1.93X10^8^ | 0.0050 | 1.2X10^-4^ |
| Healthy blood | P54N4 | 8.98X10^8^ | 0.0042 | 2.4X10^-4^ |
| Healthy blood | SX3AS | 2.11X10^8^ | 0.0032 | 1.1X10^-4^ |
| Healthy blood | UZ14A | 1.56X10^8^ | 0.0066 | 2.2X10^-4^ |
| Healthy blood | VOHG1 | 4.48X10^8^ | 0.0057 | 2.3X10^-4^ |
| Healthy blood | XM2C6 | 5.77X10^8^ | 0.0031 | 3.2X10^-4^ |
| Healthy blood | A8ZPA | 1.52X10^8^ | 0.0032 | 7.5X10^-5^ |
| ALL | KYXW8 | 1.12X10^8^ | 0.0036 | 3.4X10^-4^ |
| ALL | XAO44 | 1.81X10^8^ | 0.0027 | 7.5X10^-5^ |
| ALL | XWRN8 | 3.69X10^7^ | 0.0032 | 2.5X10^-4^ |
| CLL | 14103731 | 4.08X10^8^ | 0.0014 | 4.1X10^-5^ |
| CLL | 14103756 | 4.98X10^8^ | 0.0015 | 2.8X10^-5^ |
| CLL | 9O5HH | 8.36X10^7^ | 0.0023 | 1.6X10^-4^ |
| CLL | DQUMD | 1.53X10^8^ | 0.0014 | 1.5X10^-4^ |
| CLL | KL3AY | 5.89X10^7^ | 0.0025 | 7.1X10^-6^ |
| CLL | MVZUI | 1.08X10^8^ | 0.0025 | 1.1X10^-4^ |
| CLL | P9254 | 2.88X10^8^ | 0.0012 | 2.2X10^-4^ |
| CLL | UD8TT | 7.79X10^7^ | 0.0027 | 1.1X10^-4^ |
| MM | 53GZA | 3.60X10^8^ | 0.0018 | 2.7X10^-5^ |
| MM | 451514 | 1.27X10^8^ | 0.0030 | 4.7X10^-5^ |
| MM | 14103780 | 1.71X10^8^ | 0.0022 | 1.4X10^-4^ |
| MM | 14103813 | 6.10X10^7^ | 0.0022 | 2.5X10^-4^ |

**Table 2S**

**Quantification of 5hmC clusters by measuring photobleaching steps**

The number of fluorophores detected in each isolated fluorescence label along the DNA has a crucial role in determining the actual percentage of 5hmC in the genome. A scenario in which an isolated observable label contains multiple fluorophores, due to multiple 5hmC sites residing in a sub-resolution area along the DNA, could have a significant impact on the reliability of our analysis. Therefore, it is crucial that we validate the characteristic amount of fluorophores, and therefore the number of 5hmC sites, per isolated fluorescent label. In order to assess the number of fluorophores, we monitored the photobleaching process which fluorophores undergo when exposed to intense laser excitation. Quantifying the amount of photobleaching steps per isolated label enabled us to accurately determine the number of 5hmC sites per detected label. Over 90% of the analyzed 5hmC sites contained 1-2 5hmC labels (Figure 2S). Approximately 1000 fluorescent spots along the DNA were analysed in order to construct the distribution of 5hmC clusters. The full distribution allowed us to correct for this effect as part of the calibration procedure. Effectively, 1.45 fluorophores are present in each labeling site.


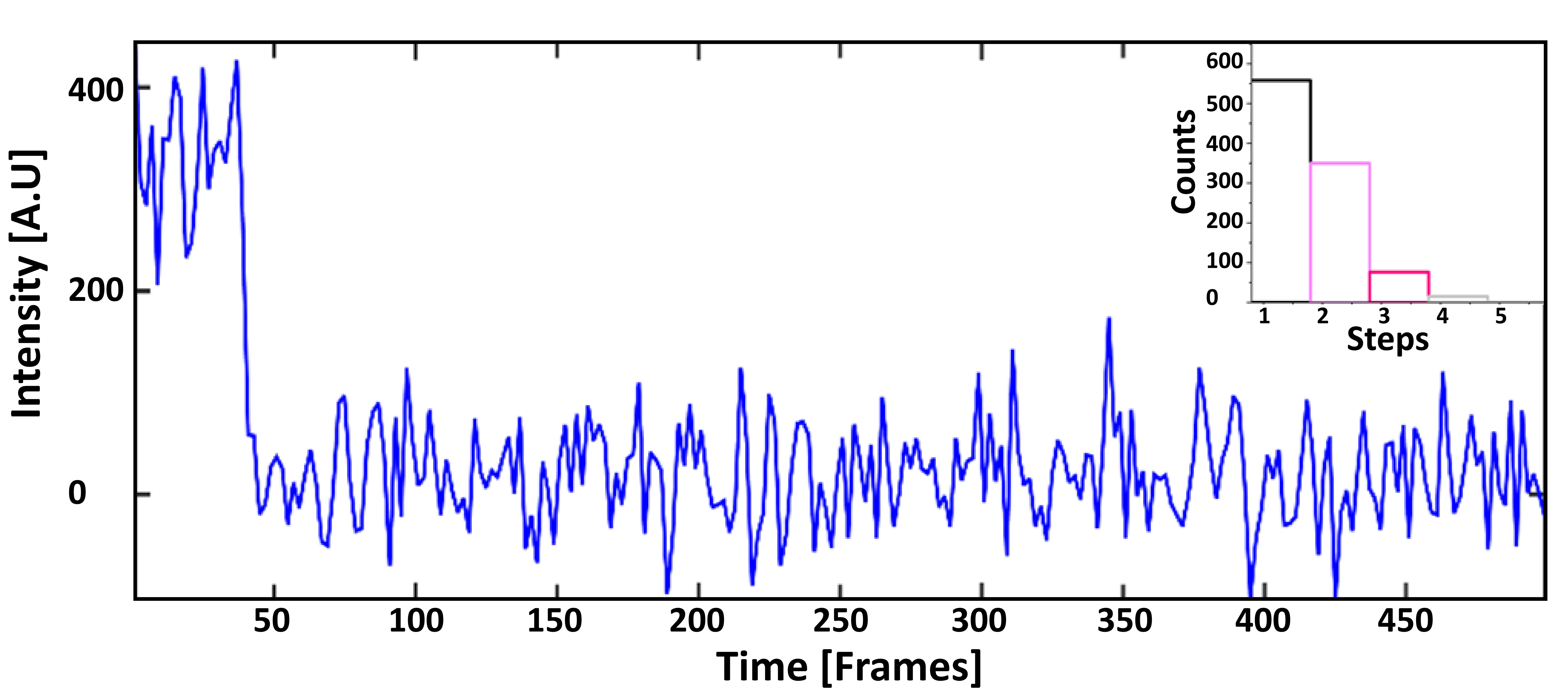


**Figure 1S. Photobleaching steps quantification.** Representative intensity time-traces of a single 5hmC fluorescent label. In-set: Distribution histogram depicting the number of photobleaching steps (5hmC residues) per label. Over 90% of the analyzed 5hmC sites contained 1-2 5hmC labels.

**Mass-spectroscopy validation**

In order to further validate our results, we used mass-spectroscopy (MS) to analyze 12 healthy blood samples (Figure 1S). Measurements were performed on a Xevo TQD instrument (Waters) according to published protocols and using 2µg of DNA per sample. The average %5hmC/dNTPs of the healthy individuals was found to be ~0.0044%, corresponding well with the 0.004% average of the single molecule data.


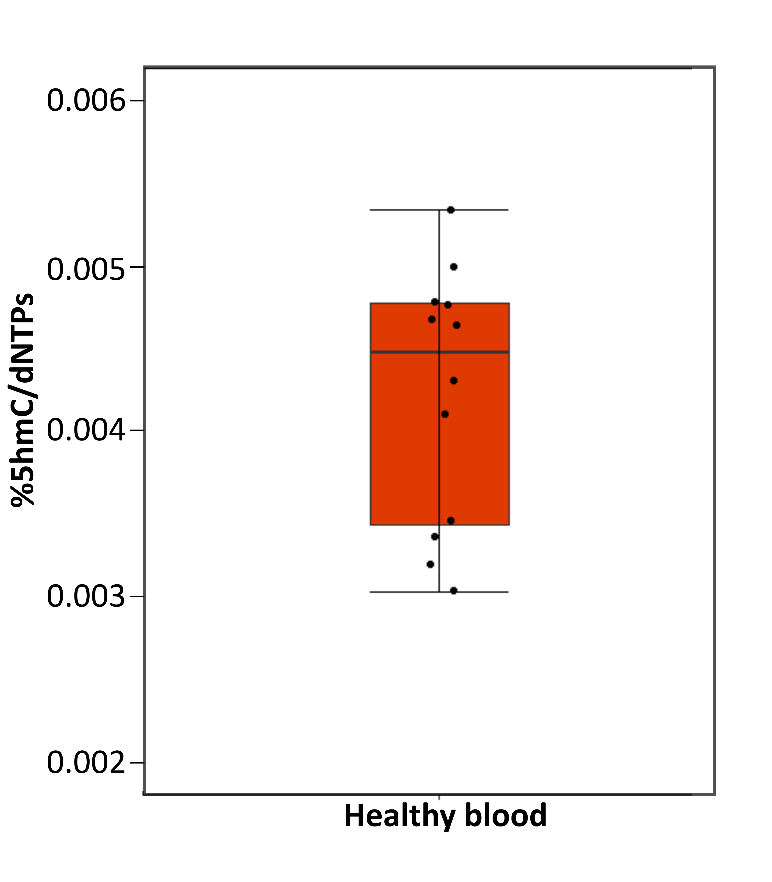


**Figure 2S. Mass-spectroscopy results of healthy blood samples.** Box plot of 5hmC level as calculated from MS results of healthy blood (*n=12*).

**Calibration and normalization**

We have included several parameters to determine the absolute 5hmC level in each sample. These comprise of the 5hmC clustering factor (defined by the photobleaching step distribution), the labeling efficiency and the DNA stretching factor over the activated glass slides. The 5hmc level for each sample was calculated by the following formula, taking into account all of the above mentioned factors:

$$\boldsymbol{\%}\frac{\mathbf{5hmC}}{\mathbf{dNTPs}}=\frac{5hmC fluorescent spots}{total length in pixels}\cdot\frac{cluster factor\cdot labeling efficiency factor}{stretching factor}\cdot100\%$$

According to our photobleacing experiments, an average of 1.45 fluorophores are present in each labeling site (cluster factor=1.45). We determined the labeling efficiency to be on the order of 84% [1] and the stretching factor is 150 bp/pixel. This stretching factor was determined by stretching a known sized DNA (lambda DNA, 48.5 Kbp) over the activated glass slides, and examining the resulting length distribution of these uniform DNA molecules.

The calculated 5hmC levels are highly correlated with the HPLC-MS results presented above.

**References**

1. Michaeli, Y., et al., Optical detection of epigenetic marks: sensitive quantification and direct imaging of individual hydroxymethylcytosine bases. Chemical Communications, 2013. 49(77): p. 8599-8601.
